# A Review on the Valorization of Macroalgal Wastes for Biomethane Production

**DOI:** 10.3390/md14060120

**Published:** 2016-06-21

**Authors:** Yann Nicolas Barbot, Hashem Al-Ghaili, Roland Benz

**Affiliations:** Department of Life Sciences and Chemistry, Jacobs University Bremen, Campus Ring 1, Bremen 28759, Germany; hashem_alghaili@yahoo.com (H.A.-G.); r.benz@jacobs-university.de (R.B.)

**Keywords:** macroalgae, biogas, industrial waste, biomethane, residues, bioconversion, eutrophication, seaweed

## Abstract

The increased use of terrestrial crops for biofuel production and the associated environmental, social and ethical issues have led to a search for alternative biomass materials. Terrestrial crops offer excellent biogas recovery, but compete directly with food production, requiring farmland, fresh water and fertilizers. Using marine macroalgae for the production of biogas circumvents these problems. Their potential lies in their chemical composition, their global abundance and knowledge of their growth requirements and occurrence patterns. Such a biomass industry should focus on the use of residual and waste biomass to avoid competition with the biomass requirements of the seaweed food industry, which has occurred in the case of terrestrial biomass. Overabundant seaweeds represent unutilized biomass in shallow water, beach and coastal areas. These eutrophication processes damage marine ecosystems and impair local tourism; this biomass could serve as biogas feedstock material. Residues from biomass processing in the seaweed industry are also of interest. This is a rapidly growing industry with algae now used in the comestible, pharmaceutical and cosmetic sectors. The simultaneous production of combustible biomethane and disposal of undesirable biomass in a synergistic waste management system is a concept with environmental and resource-conserving advantages.

## 1. Why Use Macroalgal Residues for Biomethane Production?

The rapid growth of the world’s population has increased demand for energy and food production. Fossil fuels are the current major sources of energy, providing up to 80% of the annual demand [1]. The major industrial and threshold nations, such as the U.S., Germany, Japan, China and India, rely heavily on carbonaceous combustibles to supply their domestic industries and to provide for their population [2,3]. However, dependence on fossil fuels has decreased recently, dropping from 85% in 2005 to 80%–83% in 2011 due to the development of a number of alternative renewable sources of energy, such as geothermal, wind, solar and biomass [1,4]. Since the availability of fossil combustibles is limited and their consumption strongly contributes to climate change and greenhouse gas (GHG) emissions [5,6], clean and renewable marine biomass could be an excellent contributor to the choice of alternative sources of energy. Generally, energy from biomass addresses the major types of energetic needs, providing electricity, heat and transport fuel, with the advantage of storage ability and utilization on demand. A number of terrestrial crops are widely cultivated for the production of bioethanol, biodiesel and biogas, including corn, sugarcane, sugar beets, palm oil and wheat [7]. These so-called “first generation biofuels” were strongly promoted during the last decade, but their cultivation and utilization were found to have increasingly negative environmental, social and economic impacts [8,9]. Not only does the production of these crops consume fresh water on a massive scale, their utilization as biofuels prohibits their use as an essential food source and may have a serious impact on soil fertility and biodiversity [10]. Furthermore, land clearing and emissions of harmful volatile fertilizer compounds adversely affect the environment [11,12]. These crucial aspects of renewable energy sources are often not taken into account in assessing their relative costs and benefits, due to the driving forces of product promotion and marketing (“green washing”), as well as complications in the calculation of the monetary value of environmental damage [13,14]. Social conflicts regarding land property and land tenure rights are also a side effect reported by the large-scale acquisition of farmland for biofuel production in Latin America, Far-East Asia and the African continent [15,16].

The “second generation biofuels”, produced from terrestrial waste and residual biomaterials, lower the pressure on the competition for resources. Since the production base consists of undesired waste biomaterials, it can be considered as a generation process of bioenergy with the simultaneous waste treatment of biowastes [17]. However, difficulties regarding process quality and conversion efficiency are frequent due to the resistance of the woody and lignocellulosic biomaterials to microbial conversion.

In order to overcome the issues facing the use of first and second generation biofuels, research has been conducted with the aim to switch the feedstock for biofuel production from terrestrial crops to algae [17,18,19]. Biofuels from algae are considered “third generation biofuels” [20]. Algae have other substantial advantages over terrestrial biomass, such as their growth in saltwater and municipal waste water and their lack of requirement of arable land and industrial fertilizers [21,22]. Moreover, algae generally have high growth rates and can potentially produce more biomass per hectare than terrestrial crops (e.g., sugarcane) [23,24].

Methane can be generated from thermal or biological gasification. In this review, we focus on the process of biological gasification (anaerobic digestion), which is generally the process of choice for biomass with a high water content [25]. Macroalgae, such as seaweeds, can be used in the production of biomethane, an idea that has received increasing attention in recent years [26,27,28]. With almost identical attributes, biomethane can substitute fossil-based natural gas and serves as a heating combustible or as fuel for CHP-based electricity generation [29,30]. While combustion of biogas to generate electricity is a popular practice [31], new efforts are required to establish upgrading technologies for biogas to biomethane, aiming at feeding it into the natural gas grid [30,32]. Using natural gas as transportation fuel is not common at present; however, in certain countries, including Germany and Sweden, natural-gas vehicles are a part of the streetscape, and upgraded biomethane could be used as fuel [32,33]. The conversion efficiency of watery organic matter to biogas is one of the highest of any biofuels, whether of terrestrial or marine origin [26,34]. The ability of seaweeds to absorb CO_2_, their rich carbohydrate content and lack of lignocelluloses increase their potential use for biogas production [23,35,36]. A wide variety of seaweed species can anaerobically be digested to produce energy-rich methane, which allows flexibility for the choice of biomass source [37]. Furthermore, it has been suggested that seaweed biomass could play a significant role in the biomethane market of the future, due to the increased production of marine commodities and the associated development of the seaweed production sector [38]. Seaweed industries generate a considerable quantity of marginal biowaste streams, which could present a potential use in biomethanation. Furthermore, the tapping of macroalgal biomass from beached macroalgae and seaweeds from eutrophication- or hypertrophication-afflicted marine areas offers another abundant biomass supply for use in biogas plants [39,40,41]. Using these sources of waste-type biomass for biomethanation also offers important environmental and economic benefits. Removing macroalgae from beach and marine areas supports beach management for local tourism and decreases seawater pollution caused by massive algal growth [41,42].

Shilton and Guieysse suggested in 2010 that biomethanation processes should focus on marginal terrestrial organics to relieve the competing demand for stock to produce high quality food items, industrial raw materials and biofuels [43]. It is important to emphasize that the anaerobic digestion of organic matter can also be regarded as a waste degradation process and waste management technology with a simultaneous benefit of providing energy. The microbes involved do not require “first-rate” biomass, but can utilize waste or “low-value” by-products and residual products for biomethanation. This statement can obviously be applied to marine biomass in light of its importance in the near future [44]. Biofuels from marine macroalgal wastes would be named “fourth generation biofuels” or “marine second generation biofuels”. Biomethane from marine macroalgae wastes will not revolutionize the biomethane market, but offers the chance to spread the focus of feedstock materials to defuse the current tension regarding land use competition, while simultaneously managing the accumulating biowastes. Other residues and wastes from mariculture, such as fish or shellfish wastes, should also be considered “marine second generation biofuels” and may also be suitable to generate biomethane. However, they are not the subject of discussion in this work and are excluded from the estimation of the economic relevance of marine (macroalgal) biowastes as biomethane feedstock.

The following review provides an overview of the potential of the use of macroalgae from industrial residues or eutrophication as an alternative feedstock for biogas production. It describes the context of biomass growth, chemical composition and biomethane potential and introduces some types of marine waste sources suitable for biomethanation. It also considers the additional environmental benefits and economic synergies that are relevant aspects of the utilization of these resources.

## 2. Chemical Composition

Macroalgae are classified into three major groups, brown algae, red algae and green algae, based on the optical color impressions determined through their pigmentation [22]. All of these contain high amounts of carbohydrates (up to 60%), medium/high amounts of proteins (10%–47%) and low amounts of lipids (1%–3%) with a variable content of mineral ash (7%–38%) (see Table 1 for an overview of contents of macroalgae) [45,46]. The high carbohydrate fraction includes a large variety of easily-soluble polysaccharides, such as laminarin, mannitol (brown), starch and mannan (green). Hydrolyzing these polysaccharides results in monosaccharides, such as glucose, mannose and galactose (brown, green and red macroalgae, respectively) [19,47]. These carbohydrates are easily fermentable compounds in anaerobic digestion, and their facilitated extraction allows accelerated degradation [22,26]. The quantities of cellulose and lignin, normally greatly abundant in terrestrial biomass, are generally lower in the macroalgal genera [48] due to the different structural requirements in aquatic environments [49]. The absence of lignocellulosic compounds is important to microbial decomposition and facilitates the conversion to biogas [50]. One of the main structural polymers of seaweeds is alginate, which provides both stability and flexibility for water organisms exposed to flowing water [49]. Alginate and a large number of other industrially-relevant carbohydrate compounds can be found in seaweed biomass. The hydrocolloids alginate, agar-agar and carrageenans, which are commonly used as thickeners, gelling agents or emulsifiers, are produced from brown and red algae species, such as *Laminaria japonica*, *Pterocladia* and *Gelidium* [47]. Fucoidan, a sulfated polysaccharide with pharmacological application, is extracted from *Fucus vesiculosus* [51]. Glycerol, mannitol and organic acids are extracted as value-adding byproducts and used in the pharmaceutical and comestible industries. Various other non-carbohydrate products produced from seaweeds include phenols, iodine, potash, phosphorus and proteins for human and animal nutrition originating from species, such as *Macrocystis pyrifera* and *Laminaria* spp. [52,53]. The importance of macroalgae in human nutrition is due to their high concentrations of minerals, such as calcium, magnesium and potassium, as well as glutamic acid, which make them of interest as taste enhancers. In contrast to table salt, seaweeds contain low quantities of sodium and could therefore serve as a substitute, helping to address one of the biggest challenges currently faced by the food industry: the health risks associated with excessive sodium chloride uptake [47].

Seaweed composition greatly varies, not only across species, but also in response to seasonal environmental changes [41,59,60]. During the summer, macroalgae produce higher amounts of volatile solids and sugar, whereas in spring, they generate fewer volatile solids and show a higher protein and mineral content [47]. This fluctuation in composition influences the biochemical methane potential, as was shown by Adams *et al.* (2011) in a study on the seasonal variation of composition in U.K. *Laminaria digitata* with regard to its biochemical conversion to biogas [60]. The result revealed that July material, showing the highest concentration of laminarin and mannitol, yielded the highest biomethane recovery [60]. Continuous fermentation studies performed by Chynoweth *et al.* (2002) showed a positive correlation between increasing mannitol content in the biomass and a simultaneous increase in biomethane recovery [61]. Similar results describing a correlation between increased sugar content and biomethanation performance were also observed by Jard *et al.* (2013), Adams *et al.* (2011) and Østgaard *et al.* (1993), studying the seasonality of biomethane conversion of brown algae [47,60,62].

## 3. Basics of Anaerobic Digestion and Microbial Biomass Conversion

For biogas production from marine macroalgae, the collected seaweed biomass must be subjected to anaerobic digestion (AD) in biogas plants designed for this purpose [63]. The quality of microbial biomass conversion and the composition of biomaterial are closely linked criteria regarding the conversion efficiency. Biomaterial undergoes four main successive phases during anaerobic digestion, where organic matter is transformed into methane and carbon dioxide, namely hydrolysis, acidogenesis, acetogenesis and methanogenesis [2]. The respective reactions are carried out by different groups of microorganisms in a consortium-like, almost syntrophic co-existence [2]. The efficiency of conversion is largely dependent on the hydrolysis, the slowest and therefore rate-defining phase of the whole process. During hydrolysis, organic matter is disassembled by extracellular enzymes into mono- and oligo-mers [56,64]. The subsequent steps appear equal regardless of which initial biomass was used, due to the similarity of the process intermediates. Easily degradable organic matter will undergo almost complete degradation, unlike complex and crystalline polymers, which resist breakdown [65]. Unsuitable biogas feedstocks are also substances that release factors inhibiting microbial growth and activity during degradation [66]. As stated in the previous paragraph, macroalgae contain a large proportion of carbohydrates, mainly polysaccharides, such as the structural components alginate, agar and cellulose, or the storage compounds laminarin, mannitol and starch [67,68,69]. The monomeric sugars of these components, released through saccharification, are glucose (laminarin, cellulose, starch), galactose (agar), guluronic and mannuronic acid (alginate) [22], which are all molecules suitable for the microbial metabolic pathway [2]. Microbial saccharification occurs through enzymes generated and released by microorganisms. While some of the storage polysaccharides are easier to hydrolyze due to their purpose as storage materials, structural polymers are naturally quicker to cleave. The gel-forming and depolymerizing behavior of alginate [62] increases the viscosity and decreases the surface accessibility for enzymes, while the pathway functionality of agar-hydrolyzing microorganisms is still unclear [70]. However, microorganisms in the microbial consortia of anaerobic digestion were found to generate alginate lyase [62] and numerous agarases are produced by agar-metabolizing and agar-hydrolyzing microorganisms in seawater and marine sediments [70]. Microbial degradation of such polymers is therefore possible, but depends on the microorganisms involved in the process.

Experiments on the anaerobic digestion of macroalgae have identified several factors that curb the effectiveness of microbial bioconversion. These comprise cell wall structure resistance to AD [71], synthesis of antimicrobial or toxic substances by algal cells [49] and unfavorable C/N ratios in the substrate biomass [72]. Certain seaweeds are known to produce polyphenols (phytochemicals with antioxidant activity) in high concentrations, which are released during decomposition and may inhibit the degradation process [52]. Furthermore, high concentrations of sulfur compounds [73], heavy metals and salts [74] have been reported to affect degradation [75]. Reducing the effect of toxic compounds in the digester has been realized by co-digestion schemes (dilution) [76,77] and the usage of native bacterial strains with higher tolerance thresholds [74].

The quality of degradation also depends on the type and combination of microbial inoculates used during AD. It was shown that the addition of polysaccharide-hydrolyzing bacteria and methanogenic archaea inoculate efficiently enhanced methane production [78]. The utilization of sediment inoculum of the same origin of locus as the biomass to decompose macroalgae can enhance microbial tolerance of high concentrations of heavy metals, salts or sulfur in the nutrient source and environment [74]. Jung *et al.* (2013) compiled a table with different bacterial strains degrading macroalgal polysaccharides, which could potentially serve to improve the inoculum [19]. Furthermore, it has been found that it is possible to adapt bacterial communities to tolerate higher salt concentrations and thus ensure proper substrate degradation [61]. The complexity of interactions and the co-existence of the bacterial communities in the process of anaerobic digestion was long seen as a black-box system with input and output parameters [79]. However, recent research has advanced to the microbiological scale to obtain more detailed information on biochemical interactions and also focuses increasingly on the communities’ modes of functioning during anaerobic digestion [78,80,81,82].

## 4. Biomethane Potential of Macroalgae

As stated previously, macroalgae could undergo a relatively complete conversion to biomethane due to their suitable chemical and structural composition [26]. The final yield of methane in the biogas varies from species to species and is largely influenced by the degree of optimization of the parameters in the production process, but the range is generally between 50% and 60%. Proximate estimates on biochemical methane potentials and biomethane recovery are based on substrate composition and the respective standard values for carbohydrates, proteins and lipids [83]. Methane yields from laboratory-scale experiments on AD of seaweeds have been reported in the literature since the 1970s [61,84,85] and are again the object of considerable focus in experimental studies [80,86,87] and review articles [22,26,88]. Marine biomass has shown promise for stable methane production, yielding between 140 mL and 280 mL of CH_4_ per g volatile solids (VS) for green and brown algae genera, such as *Sargassum*, *Gracilaria*, *Laminaria*, *Ascophyllum* and *Ulva* [40,73,89]. Some studies even suggest biomethane recovery of 260–500 mL CH_4_ per g VS for *Laminaria* sp., *Macrocystis* sp. and *Gracilaria* sp. [61,90,91], values that are comparable to the yields from terrestrial energy crops [31]. Table 2 shows methane yields of some seaweed species. A mesophilic temperature range for AD of macroalgae is presumed to offer a good balance between optimizing biomethane recovery and maintaining bioreactor stability [92], while a thermophilic process temperature increases the risk of bioreactor instability [93,94,95]. Typical organic loading rates between 0.5 g VS·L^−1^·d^−1^ and 3 g VS·L^−1^·d^−1^ were reported for continuous anaerobic digestion in the literature [80,95,96]. A review report by Chynoweth *et al.* (2002) stated organic loading rates up to 11.2 g VS·L^−1^·d^−1^ and pointed out the variability in performance related to the respective seaweed biomass used [61]. While the anaerobic digestion of some macroalgal biomass triggered bioreactor instability [80,95] up to total bioreactor breakdown [97], stably operating bioreactors and proper biomass degradation were also observed for other types of macroalgae [63].

New bioreactor designs were developed in order to improve the bioconversion of seaweed to biomethane, including solid-concentrating vertical and baffle flow reactors and a fluidized bed reactor. All systems were developed with the aim to increase the solid retention time (SRT) of the substrate in the bioreactor and to decouple hydraulic retention time (HRT) and SRT. This approach showed an improvement of the bioconversion performance by increasing the bioreactor solid loading [61]. Compared to the new designs, the classic continuous flow stirred-tank reactor (CSTR)-systems displayed limitations in the loading rates and system performance. Biomethanation with increased solid loading would require unacceptably large reactors, which make the process economically not viable. Small-scale plug-flow bioreactors used in the AD of animal manure are common systems in Germany. They display a similar approach of higher solid retention time and can therefore cope with higher TS contents in the bioreactor, allowing their utilization for the AD of seaweed biomass [95]. A two-phase digestion system, separating hydrolytic and methane phases, has also shown promising biomethane recovery and increased bioreactor stability when applied in AD of macroalgae. The two-phase system allows optimization of the process parameters in each of the phases, leading to an overall improvement of the anaerobic digestion process [61].

## 5. Optimizing AD and Applying Pretreatment to Improve Biodegradability

A number of strategies have been tested to improve methane yield (Table 3), which include the removal of heavy metals before production [101], mild thermal pretreatments [50], mild thermo-chemical [102] and thermo-physical pretreatments [103], co-digestion with other substrates [77], changing the microbial inoculate used for AD [78] and improving microbial tolerance towards AD inhibitors and fermentation products [77]. Harsh thermal pretreatments are often applied to biomass with high lignocellulosic content (e.g., terrestrial crops), which could result in the formation of AD inhibitors [104]. However, seaweeds lack lignocellulosic materials: hence, mild thermal pretreatments are often sufficient for their complete degradation [50,102,103]. Mechanical pretreatment methods, such as maceration and chopping, have also been successfully shown to improve biomethanation [77,105]. Such methods facilitate the liberation of sugars and increase the surface area available for microbial activity by the reduction of substrate particle size and increasing the area/volume ratio [55]. Other strategies that can be applied to improve methane recovery include pretreatment with organic acids (e.g., citric acid) and enzymes (e.g., cellulase) [37,106]. Many of these pretreatment methods facilitate the degradation process to moderate or considerable degrees. However, suitable application of the respective method is dependent on the biochemical efficiency, economic feasibility, balance of energy inputs and outputs, resource utilization and quantity of waste produced during the process [107,108]. Assessment of locally-available synergies, system analysis or life cycle analysis (LCA) can narrow down the choice of pretreatment for a particular process.

Pretreatment (PT) is a popular method to accelerate the AD, to increase the biomethane yield, making unavailable substrates accessible for microorganisms and accelerating the process of substrate conversion.

Mechanical pretreatment is applied to increase the specific surface of the biomass, to reduce the particle size and to break up the cellular structure of the biomass [22]. The advantages are the increased possibility of enzymatic attack, the reduced sludge viscosity and, consequently, a reduced agitation energy input [108]. Most solid feedstock biomaterials are mechanically pretreated before entering the AD process, and this is frequently the first step in a combined pretreatment process [105]. A potential drawback is the presence of inert materials in the biomass, such as sand, stones or metal pieces, which can cause costly damages to rotating blades or knives. However, many industrial-scale applications incorporate a combination of chopping, grinding or hammering to reduce excessive material wear out [108]. Several types of mechanical pretreatment have been shown to considerably improve the biomethane potential from macroalgae biomass [105,109].

Thermal pretreatment consists of heating the moist biomass to temperatures of typically 100 °C–190 °C, to disrupt hydrogen bonds that hold together biomass macrostructures (e.g., crystalline structures) [86,103]. The PT effect is dependent on temperature and exposure time and triggers the reduction of substrate residence time in the bioreactor by improving the biochemical conversion [22]. However, compared to the energy input for PT, thermal pretreatment is not considered efficient and is therefore often applied in a combination that is more thermo-chemically effective [81]. Usage of waste heat (e.g., from a power plant) for this form of pretreatment is a favored approach [95]. A drawback to consider is the possibility of inhibitory product formation during pretreatment with high temperature, which can lower microbial activity [108]. Thermal pretreatment of macroalgae was found to be conducted as a part of a combinatory pretreatment approach [77,103] or rather as the sole pretreatment [86].

Chemical pretreatment involves the use of harsh chemicals such as acids, bases or oxidative reagents. To improve the effectiveness of the pretreatment with chemicals, they are usually used in combination with temperature, such as the thermo-chemical pretreatment [110,111]. In particular, thermo-acidic pretreatment has shown a positive effect on the improvement of the biomethane potential from manure and lignocellulosic material [111], as well as for macroalgal biomass [56,102,112]. Thermo-acidic pretreatment enhances the saccharification of polysaccharides and, therefore, also carbohydrate-rich seaweed biomass [56,102]. However, temperature and acid concentration ranges must be carefully selected, since insufficient disruption potential will lead to a low improvement of biomethane potential [102], while excessive use may severely lower the microbial conversion efficiency and therefore also its biomethane potential [108].

Biological pretreatment can be applied in the form of enzyme addition, fungi or aerobic bacterial pretreatment or as an anaerobic pre-acidification step in a two-stage anaerobic digestion system [108]. The latter is commonly practiced in industrial-scale anaerobic digestion of terrestrial biomass, while the treatment of biomass with lactic acid bacteria (LAB) is also applied, in the form of biomass ensiling. Ensiling also allows macroalgae to be stored for an extended period of time without undesired biomass degradation [82].

Combined processes, such as steam explosion, thermo-acidic or thermo-mechanical pretreatment, are often selected, due to the fact that one single pretreatment method does not provide the desired results. They are generally more effective at improving the biomethane potential, but also more complex and resource-demanding. Applying thermo-acidic pretreatment and steam explosion on macroalgal biomass has led to impressive improvements on biomethane yields and biomethane conversion [86,102,103]. Other pretreatment methods, such as ultrasound treatment, electrokinetic or pressure disruption, also exist, but are applied to liquid materials, e.g., in sludge treatment (e.g., sewage sludge or digester sludge).

To conclude, there is no universal, best-performing pretreatment recipe; the success of pretreatment application depends on the type of biomass combined with the pretreatment method(s), the availability of resources (e.g., waste heat) and the employed technical setup (e.g., reactor design). For a successful pretreatment application, economic aspects must be considered, as well as the technical feasibility. In some cases, no pretreatment is need at all to achieve successful AD.

Biochemical process optimization mainly aims to support microbial substrate degradation by the addition of substances that assist the metabolic activity and proliferation of microorganisms. Any deficiency of essential nutrients during degradation may strongly decrease the process performance [120]. To address this, macroalgae have been subjected to co-digestion with sewage sludge [76], cattle manure [77] or carbon-rich organic waste [72,103]. These additives serve to optimize the feedstock C/N ratio and thus improve bioconversion [2]. Nutrient ratios of the substrate combined with different substrate retention times within the digester are important parameters that influence the quality of the bioconversion. Nutrient limitation or reduced nutrient availability for the microorganisms can lower the biodigester performance. Ratios of 15:1 for C/N and 75:1 for C/P were shown to be non-nutrient-limiting for the digestion of *Macrocystis* [61]. The co-digestion of macroalgae with glycerol, secondary sludge or waste-activated sludge increased methane yield between 18% and 26% [87,121].

The majority of studies on the biomethanation of macroalgae have produced promising results only at the laboratory scale, with few pilot-scale studies published to date [63,122,123]. However, the availability of various types of seaweed biomass and diverse options to improve the AD process make marine macroalgae an interesting candidate for larger scale biogas production.

## 6. Growth Conditions of Macroalgae

Marine macroalgae are among the most ubiquitous organisms on the planet. They are mostly found in coastal aquatic environments [124] and compose approximately 50% of global biomass [125]. About 71% of the Earth’s surface is covered with saltwater, which provides the ideal habitat for the abundant growth of marine macroalgae [126]. Seaweeds can be found down to 150 m beneath the ocean’s surface [124] and can grow either on solid substrates, such as rocks, wood and shells, or freely floating on seashores, in salt or brackish waters [127]. The natural habitats and growth conditions of seaweeds vary depending on the species. The main environmental factors that determine their occurrence, abundance and growth are water temperature, sunlight irradiation, availability of CO_2_ [128], salinity [129], hydrodynamics of the locus [130] and the presence of necessary nutrients through riverine fluxes [131].

Optimal growth temperature ranges from <15 °C (*Ascophyllum* spp. found in the Northern Hemisphere in sheltered areas) through 15 °C–20 °C (*Gelidium* spp. found in rocky areas at depths of 2–20 m) to 20 °C–25 °C (*Ulva pertusa*, a species from the Japanese coast line) [132,133,134]. Interestingly, seaweeds can adapt to temperature fluctuations, including high temperatures, in order to achieve maximum growth rates [135,136,137].

Sun irradiation, coupled with light intensity, plays an important role in macroalgal proliferation. Sublittoral species, permanently covered with water, such as *Laminaria saccharina*, show ideal growth at a low photon flux density (30–70 µE·m^−2^·s^−1^) and suffer from growth inhibition at high photon flux density (250 µE·m^−2^·s^−1^) [136,138]. Eulittoral species in the intertidal zone, naturally exposed to higher irradiation, such as *Ulva lactuca* or *Porphyra umbilicalis*, are only slightly inhibited by high photon flux densities [138]. Commercially-used seaweeds, such as *Kappaphycus* and *Eucheuma*, grow best in bright light [134].

Water salinity affects the chemical composition and the diversity of seaweeds [136,139]. *Vaucheria* and *Rhizoclonium* species can tolerate only low salinity levels of 10‰ parts per thousand (PPT), which is the usual condition of brackish waters [136,140]. Intertidal seaweeds can tolerate up to a 100‰ salinity concentration [136,139]. A concentrated saline environment also leads to correspondingly high concentrations of mineral sodium and potassium compounds in the macroalgal biomass [46].

The macroalgal carbon source is inorganic carbon dioxide, which is used in the Calvin cycle to produce diverse types of organic compounds. It is mainly present in the form of dissolved HCO_3_^−^ with an aqueous concentration more than 50-times that of the Earth’s atmosphere [23]. The uptake rate of CO_2_ by macroalgae ranges from 100 to 2232 µmol CO_2_·g^−1^ (dry wt.)·h^−1^ with the highest rates for *Enteromorpha compressa* (green), *Sargassum muticum* (brown) and *Porphyra yezoensis* (red) [23].

The presence of dissolved nutrients in the water significantly influences the quality and quantity of macroalgal growth, with nitrogen, phosphorous and iron being the most important elements and often among the limiting growth factors [58]. Naturally, nitrogen compounds, such as nitrite or nitrate, reach the marine environment through discharge in run-offs and river flows, while phosphorus and iron release are biologically catalyzed by rock weathering [141]. At present, the marine influx of nitrogen, phosphorous and iron is supplemented by anthropogenic discharge, causing local accumulation in marine areas [40,74].

Water currents and waves facilitate the advection and diffusion of dissolved nutrients and gases, as well as the transport of sediments and spores [142]. Macrophyta proliferate attached to substrata and are exposed to shear stress through water movement. Excessive or critical shear stress will eventually lead to the erosion of sediments, leading to macroalgal detachment: the product is classified as particulate non-living matter [143]. Areas exposed to extreme current and wave movement are generally inhospitable to many macroalgal species. Other factors that affect the growth of macroalgae include biotic interactions with other organisms (e.g., competition with other growing macroalgae: this effect plays an important role in eutrophication events) [136].

## 7. Occurrence of Marine Eutrophication and Improvement of Coastal Management

Seaweeds growing naturally on seashores can provide abundant stock for biofuel production. The sea-borne biomass is washed ashore after strong inshore wind and tidal activity (e.g., during storm periods) [144]. Furthermore, nutrient-rich lagoons or estuaries present an excellent growth pool for macroalgal biomass [74,96]. Algae that remain on the beach and decompose can negatively impact the coastal ecosystem [145] and local beach tourism [146]. Some beach areas note a continuous supply of wild beach algae washed ashore throughout the year with seasonal variations in biomass quantity and quality [47,60]. However, the exact amount and composition of the biomass is difficult to predict [41]. Incidents of extreme macroalgal proliferation and accumulation as a consequence of favorable growth conditions are called algal blooms and refer to marine eutrophication [147]. This is a natural phenomenon, which lowers excessive phosphate and nitrate concentrations in eutrophied waters. Macroalgae act as biofilters and fix the dissolved compounds in the form of biomass [6,148]. However, problems may arise with reference to the scale and location of this process [41]. This happens when macroalgae washed ashore degrade rapidly, creating greenhouse gas (GHG) emissions and foul odors [123]. Such odors are unpleasant and hazardous to health and the environment [149], in particular when they arise due to harmful algal blooms (HAB) [145]. The biomass is either removed through a costly process, confined to landfills or, most often, dumped and improperly disposed of [73]. *Ulva* is classified among the organisms responsible for the occurrence of HABs, as they pose a threat to the environment, public health, fisheries and economies [150]. Opportunistic macroalgae present a growing environmental problem in many coastal zones worldwide [41,145,151].

**Figure 1:**
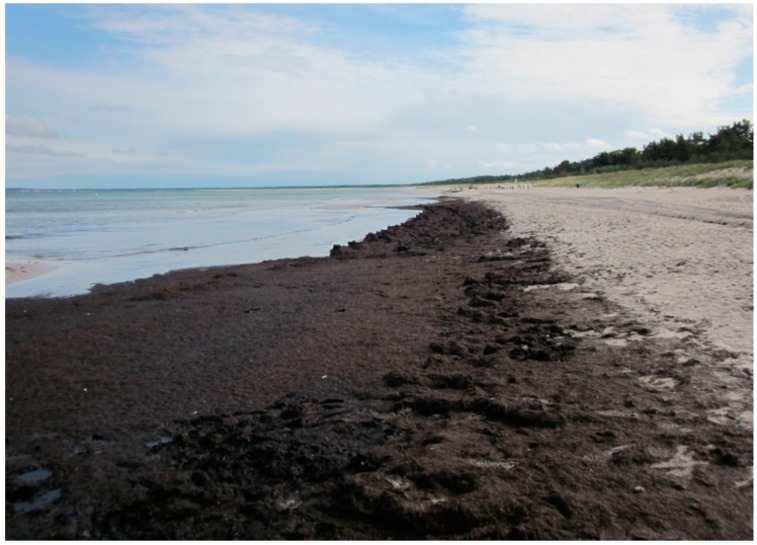
Macrophyta accumulation at Juliusruh beach in Rügen, Germany (54° N, 13° E), on the Baltic Sea shore. Macroalgae were harvested in August 2011. Photo: Yann Barbot 2012.

Eutrophication occurs during intensive irradiation, typically during the summer, which provides solar irradiation for photosynthesis and warms up near-surface water regions. Windless conditions, calm seas and nutrient availability are other factors that may combine to create perfect hydrodynamic conditions for fast biomass proliferation [149,152]. River estuaries, shallow basins, coastal lagoons and semi-closed waters are therefore most susceptible to eutrophication [153]. These areas also restrict the discharge of nitrate- and phosphate-enriched water into the open sea, causing local accumulation [40]. Figure 1 shows an example of the accumulation of macroalgae on the beach of Juliusruh in Rügen, Germany as a consequence of eutrophication. Eutrophication events are regularly reported in Venice Lagoon (Italy) [96], Orbetello Bay (Italy) [74], Brittany (France) [73], Qingdao beach, the Gulf of Mexico [154], areas near Sopot beach (Poland) [155] and other locations in the Baltic Sea as a semi-enclosed sea [153,156]. Orbetello lagoon in Central Italy receives around 5000 t of marine macroalgae annually: these are washed ashore only to be removed at considerable expense and dumped in landfill [74]. Brittany’s coast is affected by eutrophication because it provides favorable conditions for macroalgal growth, including a rocky foreshore (attachment), wide tidal range (rinsing), water transparency (irradiation), nutrient availability in the water and renewal by tidal currents and stirring of the medium (nutrient availability) [47]. In 2011, the disposal of 100,000 t of *Ulva* from Brittany’s coasts was ordered to mitigate its impact on local tourism: the costs of disposal ranged from US$ 10–150 per ton [154]. A quantity of 50,000 m^3^ macroalgae is harvested every season in Venice lagoon to counteract eutrophication [76].

Nutrient enrichment is one of the key factors enhancing massive macroalgal growth [40]. The situation is aggravated by increased nutrient enrichment from estuarine farmland sustaining intense fertilizer application (nitrogen, phosphorous and potassium), as well as urban and industrial discharges [156,157]. Events leading to extensive eutrophication are called “hypertrophication”, and biomass resulting from such events is considered harmful, as well as unpleasant. In 2008, a large green tide invaded the beaches of Qingdao in China, obstructing the area in its capacity as the venue for the sailing events of the Beijing Olympics. Over a million tons of *Ulva* biomass had to be removed, involving 10,000 people and incurring costs of US$ 30 million, excluding the economic losses of local aquaculture operations [154]. Eutrophied macroalgae may present a marginal biomass source for various industrial applications [158] including as potential feedstock for biogas production [39,102]. The potential of “green, brown or golden tides” for biomethanation has already been studied by several research groups and has been evaluated for the Irish and Baltic biofuel market [17,40,41].

Beyond its role in providing a raw biomaterial for the biofuel market, the removal of algal biomass in hypertrophication-affected areas would greatly contribute to the general improvement of environmental conditions in these coastal areas [6,159]. Cleaning aquatic environments of pollutants (environmental remediation) changes negative environmental externalities, ultimately leading to a positive outcome [17]. The growth of seaweed biomass in during an eutrophication event reduces aqueous nutrient levels and permits the recycling of relevant carbon, nitrogen and phosphorus. The possible provision of phosphorus is of particular interest for agriculture, as traditional stocks, such as geological potash deposits, are becoming scarce due to depletion [6]. Seaweeds are also known to remove contaminants, such as heavy metals, from polluted sediment and surface water areas [74]. This fact could also be applied in the remediation of contaminated coastal areas polluted by industrial effluents [160].

The collection or harvesting of marine macroalgae from aquatic environments requires efficient, simple and affordable methods. Marine macroalgae can be harvested in different ways, either manually or mechanically, depending on the biomass linkage and whether they are accumulated on the beach or floating in open or shallow waters [53,146,161]. Macroalgae cultivation facilities represent a source of experience regarding macroalgae harvesting from water. Despite the large-scale production in Asian macroalgae-producing countries, much of the harvesting is done manually due to the low costs of manual labor. In the coastal areas where access is easy, surface floating seaweeds are simply harvested by raking and hand picking while they are loaded onto small boats [162]. Mechanical harvesting is a cost-effective method that is used in Europe due to the technical feasibility. In Ireland, *Coralline officinalis* and *Saccharina latissima* are harvested mechanically. France and Norway harvest *Laminaria digitata* and *Laminaria hyperborea* mechanically by specialized boats, which are equipped with vacuum-suckers, rotating blades and trawls and can accommodate relatively large quantities of seaweeds [53,162,163].

Macroalgae accumulated on beaches initially present an easier prospect for harvest, due to their occurrence in piled and packed stacks. However, there may be an associated intake of larger quantities of sand, presenting an issue for further biomass processing and increasing the cost of biomass transportation [63]. There are techniques to separate sand and biomass [164], but these require a further indispensable and cost-increasing step in the supply chain. Accordingly, techniques of harvesting beached macroalgae without mixing in large quantities of sand are advantageous. This could be done through elaborate biomass pickup or on-site “sea-washing” before loading [161]. Due to the novelty of the problem, experience from standardized management concepts of cleaning beaches from macroalgae cannot be transferred. Current strategies incorporate a variety of local technical and organizational solutions, such as the patchwork solutions on the Baltic Sea shore in Germany [146], the clearing of eutrophied beaches in Brittany (France) [42] or the manual cleaning of the Olympic regatta venue covered in 600 m^2^ of *Porphyra yezoensis* in Qingdao in 2008 [154,165].

Generally, the development of new macroalgae harvesting techniques and machines is in demand, but new developments should also be approached with care. Concerns have been raised regarding industrial harvesting techniques, which might lead to endangering the habitats of sea life and birds through the removal of coastal kelp, naval transportation traffic and raking or swirling up the seabed during mechanical harvest [163].

## 8. Prediction of Macroalgal Growth Using Satellite Imagery

Based on the existing knowledge of the ideal conditions for the growth of macroalgae, it may be possible to predict the potential sites of their growth using real data and climate simulation models [138,143,166]. Satellite imaging is a suitable technology to support the early detection and monitoring of algal blooms and growth. It can be used to determine the affected area size, approximate biomass quantity and types of organism involved [167]. Satellite imagery allows the regular observation of environmental changes in quantities such as chlorophyll concentration, sea surface temperature, marine CO_2_ concentration and net radiation [168]. Such data, combined with meteorological and hydrodynamic datasets, could also be used for designing simulation models in order to predict the location of predominant algal growth [169]. Together with laboratory and field data, such models can provide an insight into the potential growth rate of seaweeds in various aquatic environments and narrow down the timeframe and spatial location of potential seaweed accumulation [170].

## 9. Macroalgal Biomass Obtained from Industrial Wastes

**Figure 2:**
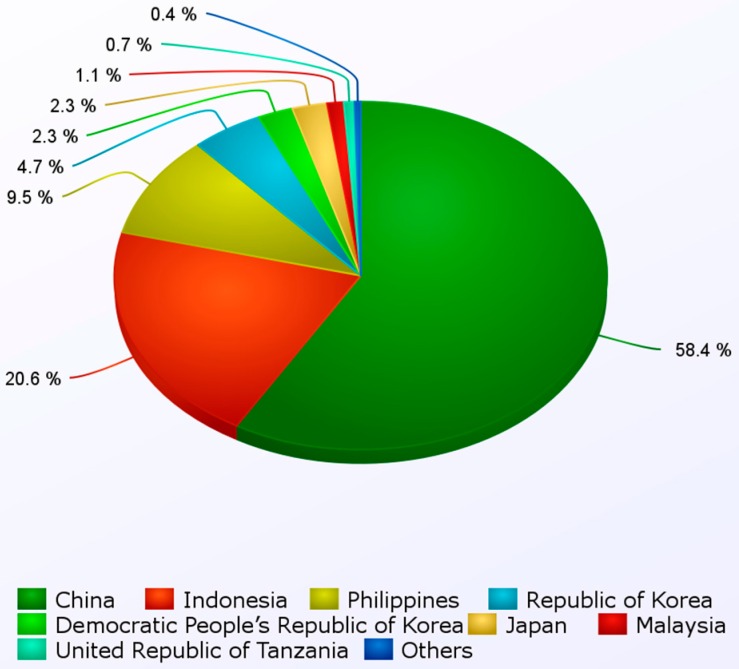
Worldwide production of seaweeds in 2012. Data by the Food and Agriculture Organization of the United Nations (FAO) in “The State of World Fisheries and Aquaculture”, 2012 [44].

Due to increasing interest in the cultivation of macroalgae for the production of foods and other valuable products, the total volume cultivated reached nearly 19 million tons (worth US$ 5.7 billion) in 2010 alone (Table 4). In Asian countries, such as Japan and Korea, the most commonly-cultured seaweeds include *Porphyra, Undaria pinnatifida* and *Saccharina* (*Laminaria*) *japonica*, which are used for human consumption [171]. Almost 90% of the industrially-cultivated seaweeds originate either from China, Korea, Japan, Indonesia or the Philippines ([44]; Figure 2). However, successful cultivation has also occurred outside Asia, with significant yields in Mexico, France, the U.S. and Norway [171]. It is estimated that the western seaboard of South America, the north Atlantic Coast of North America and the European coast could be utilized for industrial macroalgae cultivation [26]. Therefore, new cultivation and harvesting approaches are under development to ensure successful and enduring cultivation schemes [172].

In addition to the direct consumption of macroalgae as a food source, they are utilized for the production of industrially-valuable products such as alginate, carrageenan, agar, bioplastics, dyes, cosmetics and pharmaceuticals [134]. France and Norway are the main European producers of seaweed, with annual harvests between 50,000 and 120,000 t, mainly comprising *Laminaria* spp. for hydrocolloid production [47]. On average, *Laminaria* is composed of 26% ash, 23% alginate, 14% laminarin, 12% mannitol, 12% proteins, 6% cellulose, 5% fucoidan and 2% lipids, which sums up to 60% fermentable sugars (from dry weight) [53]. Assuming that algal hydrocolloids, such as alginate, are extracted and none of the remaining compounds are utilized elsewise, this leaves 51% of the remaining organic residues as possible fermentable waste.

In general, factories produce a considerable volume of waste byproducts in the processing of macroalgae (see Table 5 for an overview). Current usage of seaweed wastes includes, but is not limited to, the production of fiber, glycerol, biofertilizers and organic acids [22,173,174]. Furthermore, waste seaweed obtained from the alginate industry is also used for the removal of toxic heavy metals, such as copper, zinc and cadmium [175]. The bioremediation of contaminated aquatic areas and waste waters using macroalgae as an adsorbing agent has already proven successful [176,177]. Macroalgae are known to contain up to 30%–40% alginates [134]. The extraction of these biomaterials from seaweeds on an industrial scale generates considerable algal waste material, which could be used for further applications. Nearly 39,000 tons of alginate and 28,000 tons of carrageenan are extracted annually worldwide [58]. The potential of utilizing industrial seaweed wastes for the production of biomethane has already been tested, with promising results. Edyvean *et al.* (1988) produced 320 L·kg^−1^ VS biogas with 62% methane from seaweed waste following alginate extraction in the course of a 20 days’ retention time [178]. Anaerobic digestion of waste sludge obtained from alginate extraction from *Laminaria hyperborean* and *Ascophyllum nodosum* successfully yielded between 0.10 and 0.15 L·g^−1^ VS and 0.07 and 0.28 L·g^−1^ VS biomethane in the course of a 16 days’ retention time [179]. Furthermore, Carpentier *et al.* (1988) used waste flotation sludge from the industrial alginate production industry for biomethane conversion, obtaining a biomethane yield of 270 mL·g^−1^ VS [180]. Furthermore, Barbot *et al.* (2015) showed that biomethane recovery from industrial residues of *Laminaria japonica* as the substrate biomass yielded 173 mL·g^−1^ VS in stable continuous single digestion over three hydraulic residences. These laboratory results were followed by a pilot-scale trial in a 1.7-m^3^ pilot plant system, showing the feasibility of process upscaling [63]. The study was initiated based on the fact that 50,000 t (wet mass) of *Laminaria japonica* waste biomass are generated annually by the Haizhibao Ocean Science and Technology Co. Ltd., Shandong, one of the largest producers of algal food products in China [63]. Other types of industrial waste materials include the residues of agar-agar production (“macroalgae meal”) from *Gelidium sesquipedale*. One of the largest world producers of agar-agar, an industry located in northern Spain, generates 2–2.4 t of dry macroalgae meal per day [181].

In general, the composition of macroalgal residues is diverse and, in particular, depends on the macroalgae composition and the type of compounds extracted during the industrial processing. It might be presumed that processed seaweed residuals contain considerably less volatile solid matter than native seaweeds and therefore reduce the biomethanation potential. In fact, the VS content of the macroalgal waste is only slightly lower than that of other biowastes and similar to that of common manures [186]. Agricultural wastewater runoffs are another unexploited potential source of nutrients for macroalgal cultivation, while allowing recovery from industrial wastes; its use as a cultivation medium has been demonstrated [187]. While industrial seaweed waste is an underestimated, untapped and promising source of biomass, more research is still required in order to identify suitable substrate types and the respective appropriate conditions to maximize biomethane production. Processing of the feedstock biomass in the form of conditioning or preparation might present a necessary step before proceeding to microbial biomethane conversion. Further, in many processing industries, such as compound extraction of alginate or agar, marine biomass disintegration is a central part of the product manufacturing process [178,179,180,181]. By definition, these respective residues will be recovered in a fractured and disrupted condition, which presents an advantageous state for further microbiological conversion.

## 10. Economic Aspects and Relevance

The economic aspects of macroalgal wastes-to-biomethane can be divided into two phases: (1) providing the biomass, including harvest, reconditioning and transport to the site of conversion; (2) bioconversion, biomass storage, refinement of end-product and treatment of wastes [61]. Economic feasibility will depend on individual cases, but the steady availability of macroalgal wastes and the logistics of biomass transportation to the biogas plant will be important aspects, as is also the case for the seaweed-to-bioenergy case. The bioconversion of the macroalgal biomass itself has shown promising biomethane yields and does not seem to present constraints. Biogas can be directly converted to electricity or upgraded to biomethane, which is an established technique and now practiced at an industrial scale [32]. The treatment of digestate and its further use as a nutrient source have also found economic interest in light of the need to transition to a circular economy and conserve resources [188,189]. While industrial residues are generated in continuous amounts and with a similar biomass composition, linked to the production process, biomass from eutrophication shows a much higher variation in composition and a high fluctuation in availability. Utilizing the latter source will necessitate biomass storage to assure a steady supply of biomass for the AD process. Recent works have explored ensiling the macroalgal biomass as a storage option [82].

Biomethane from macroalgal wastes should not be confused with the numerous endeavors to exploit seaweed biomass for the generation of biomethane, biohydrogen or bioethanol as an all-encompassing substitute for fossil-based fuels. To claim that biomethane from marine macroalgal waste will revolutionize the bioenergy market would not be a realistic approach to the topic. Instead, it should be regarded as a further step towards small-scale bioenergy production units, on-site waste treatment and a technology to improve local environmental conditions. In line with current proposals for a decentralized infrastructure of small- and medium-sized biogas plants to supply electricity, heat and upgraded biomethane [190,191], the biomethanation of macroalgal biowastes and eutrophied-borne seaweeds should be conducted in the vicinity of their location of origin.

The potential of biomethane production from industrial macroalgal biowastes is mainly restricted to the geographic regions with significant seaweed cultivation or manufacturing industries. Around 24 million tons of seaweeds were produced globally in 2012 [44], mainly brown seaweeds, such as *Kappaphycus*, *Eucheuma*, *Laminaria*, *Porphyra* and *Undaria* [44,171]. As well as the large production sites in Asia, seaweed cultivation is gaining popularity in other parts of the world, particularly Europe [18,57,192,193]. To enhance the economic feasibility, the biomass is mainly used to extract higher value compounds for the pharmaceutical, cosmetic or medicinal industries, as well as for use as animal feed supplements [46,53,57,194]. The use of seaweeds for bioenergy generation has been discussed in many scientific works, but has not been extended to industrial-scale applications, mainly due to the high costs of biomass production [18,195]. In particular, European research in upstream (cultivation) and downstream processing (industrial extracts, conversion to bioenergy) of macroalgae has intensified in recent years [47,57,192,194]. Macroalgae industries are growing worldwide to meet the increasing market demand for macroalgal products.

Globally, *Laminaria japonica* is one of the most frequently-cultivated seaweeds with a production quantity of 5.5 million tons of fresh farmed biomass [44]. Around 10%–30% (20% in average) of this biomass remains as residual biomass during harvest and downstream processing [63]. The biomethane recovery from one ton of fresh *Laminaria japonica* residue biomass (approximately 20% total solids) can be estimated at 20 m^3^ [63]. Hence, the total potential from *Laminaria japonica* residues accounts for 22 million m^3^ of biomethane per year. Similar calculations can be done for the nine million tons of *Kappaphycus*/Eucheuma production in Asia or the 50,000–120,000 tons of European *Laminaria* seaweed harvests per year [44,74]. Assuming that residual quantity and biomethane potential of macroalgal waste are similar for all cultivated, harvested and processed species, 24 million tons of seaweeds would generate 96 million m^3^ of biomethane per year. Barbot (2010) calculated that a quantity of 1700–2000 tons of dry seaweed biomass (around 8500–10,000 tons fresh) would be necessary to operate a small-scale biogas plant supplying 75 kW_el_ CHP per year [196]. With a biomass quantity of 50,000 tons of fresh *Laminaria japonica* waste generated annually by a large producer of algal food products in China, a number of 5–6 small-scale biogas plants can be continuously operated, providing a total electricity output of 375–450 kW_el_ [196].

The potential of eutrophied seaweeds is more difficult to estimate, since there is no global data available for their quantities. Furthermore, many species and species mixtures can be involved in the formation of green and golden tides [154], which makes it difficult to define biomethane recovery. The literature only contains descriptions of the quantities of locally-identified and single eutrophication events. The largest count was provided by Charlier *et al.* for the Brittany coastline in France, with an annual estimate of 100,000 tons of seaweed washed ashore, mainly *Ulva* species [154]. Furthermore, the massive green tide on the Qingdao coastline (China) in 2008 stated an estimate of one million tons of fresh *Ulva prolifera* removed from the shores [165]. Fresh *Ulva* has a volatile solid content of about 11% and a biomethane potential of 180 m^3^ per ton of VS [40]. Based on this information, the biomethane potentials are around 2 million m^3^ and 20 million m^3^ of biomethane for the biomethanation of *Ulva* from the Brittany and the Qingdao coast, respectively. There are many other reports from different European coastal areas, recording eutrophication events of beached macroalgae on a regular basis [40,41,74,76].

The costs of providing macroalgal waste biomass or marine eutrophication biomass is difficult to estimate due to the great differences in quality, quantity and availability. Considering that these types of biomass are classified as waste material or harmful biomass, the price of acquisition must be considerably lower than that of cultivated seaweed biomass. Industrial residues can be used as an additional step in downstream processing to obtain an energetically-valuable by-product before the treatment of the biowaste. This would mainly concern producers of macroalgal commodities with a respective production of macroalgal waste generated during processing, who could operate a biogas plant as a waste treatment unit at the end of their production cycle. The advantages are a continuous, free supply of biomass with a steady quality of composition.

Beached macroalgae are usually seen as unpleasant, particularly in tourist areas. Resorts and local authorities pay considerable sums to clear beaches and near-shore shallow waters of this biomass to ensure profitable tourism [146,165]. Examples in the literature state costs of 38 € per meter beach and year for clearing beach wrack in the Baltic Sea [146], 120 € per ton and year for the disposal of beached seaweed from eutrophication in the Mediterranean Sea [74], US$ 30 per ton for the removal of one million tons of eutrophication-generated seaweeds on the coast of Qingdao [154] and 6–120 € per ton for the removal of eutrophication-generated *Ulva* along the coasts of Brittany [73]. The authors of this review article identified costs of 85 € per ton for providing dried and pourable biomaterial from beached macroalgae from Rügen, Germany, based on information from one of their previous works [95]. This processed biomaterial could be readily used for the generation of bioenergy; this has been explored for the automotive industry [164]. In light of this situation, the expenses for providing biomass can be lowered while providing the service of clearing beaches of unwanted biomaterial.

Valuable by- or co-products accumulating during the production and processing of the biomass can lower the overall costs for seaweed-to-bioenergy and can add an important exploitation path to the process [61]. In the present case, this can also account for biomethane from eutrophication biomass, where extracts can be separated from the biomass prior to biomethanation. Some of the possible by- or co-products can be found in Table 6 of this article. Kraan (2013) presents a list of high-value by-products, including their market prices, which can be obtained from seaweeds, such as alginate, potash, iodine and protein or lipid feed [6]. While industrial residues are themselves by-products, biomethane from industrial residues is likely to present the last step in the downstream production process. Further product exploitation is therefore unlikely. Commodity-containing digestion effluents can also present an interesting source of by-products [61,106]. Macroalgae are known to contain high amounts of phosphorus and other nutrients necessary for crop growth. These nutrients are also found in the digestate of anaerobically-digested seaweed. In light of the increasing scarcity of available phosphate rock used in agricultural fertilizer production, biomass generation seems an interesting method of utilizing this nutrient flow [197].

## 11. Conclusions

The availability of marine macroalgal waste at a high biomass quantity level, their ready availability and their good degradation potential make them a promising choice for biomethane production. With proper management strategies, marine macroalgal waste could offer an important contribution to the sustainable supply of biomass for biogas production without excessive negative impacts on the ecosystem and avoiding fuel-food competition. Furthermore, the increased interest in macroalgae as a source of industrial raw materials and food products and the subsequent increase in cultivation predict the availability of a variety of macroalgal biowastes in sufficient quantity as feedstock for biomethanation. This approach also appears conducive to accompanying applications such as bioremediation and waste disposal, adding important environmental benefits. However, further work remains in the form of assessing the marine biomass waste market for suitable biomass sources, industrial-scale AD applications and the optimization of the biomethanation process efficiency. The process of accessing a suitable beached macroalgae feedstock could be facilitated by the use of supporting technologies, such as satellite imagery and climate simulation models, allowing the prediction and localization of the quality and quantity of eutrophic biomass sources.

Concerning the microbial conversion of macroalgal residues and waste biomass to biomethane, laboratory trials allow a preliminary assessment of the suitability of various conditions. If efficiency is found to be suboptimal, the contributing factors must be identified for each process individually, and various remediating measures may be taken. Reduced bioconversion efficiency is linked to the composition and macrostructure of the respective biomass. Feedstock containing antimicrobial or toxic substances, unfavorable compound ratios or recalcitrant organic molecules may inhibit the microbial substrate conversion. Various methods, such as pretreatment or co-digestion, are available to counteract or circumvent these issues to successfully exploit the respective biomass source for biomethanation.

Incorporated into the concept of a circular economy, macroalgal wastes from industry or eutrophication could find a niche in serving as substrates for biomethanation. The global demand for all kinds of macroalgal products is on the rise, as are numerous repeatedly reported eutrophication events. Treatment pathways of the accumulating waste biomass should be developed in parallel to the macroalgae market in order to support a circular economy and environmental remediation. This also applies to the residuals generated during anaerobic digestion of macroalgae, such as the fermentation digestate. If not suitable for processing to terrestrial fertilizer due to the high concentration of salts, it could find fertilizer application in marine aquacultures.

## Figures and Tables

**Table 1:** Composition of macroalgae (green, red and brown) regarding carbohydrate, protein, lipid and ash content. A selection of industrial use and industrial extracts are also listed based on the composition.

Compound	Green Algae	Red Algae	Brown Algae	Reference
Water content (from fresh mass)	70%–85%	70%–80%	79%–90%	[54]
Ash	18%–53%	26%–48%	33%–55%	[47]
Total organic	47%–82%	52%–74%	44%–66%	[47]
Carbohydrate	25%–50%	30%–60%	30%–50%	[19]
Polysaccharide	Alginate Cellulose Mannan Starch Ulvan	Agar Alginate Carrageenan Cellulose Lignin	Agar Alginate Carrageenan Cellulose Fucoidan Laminarin Mannitol	[19,55,56]
Protein	12%–13%	10%–16%	7%–12%	[47]
Lipid	2%–3%	0%–3%	0%–2%	[47]
Industrial extracts	Sulfated galactan, vitamins (e.g., C), antiviral and anticoagulating agents	Sulfated galactan, vitamins (e.g., C, B), mineral nutrients (e.g., iodine), agar, phycobiliproteins	Fucoidan, fucan hydrocolloids (alginate, carrageenan, agar-agar) polyphenols, mineral nutrients (e.g., iodine), pigments	[19,47,53,57,58]
Industrial use	Human food, food supplement, medicinal use	Human food, pet food production, thickener, emulsifier and gelling agent in industrial and lab use and for cosmetics	Human food, animal feed, alginate for textile printing, medical fiber, paper industry, cosmetics, agar as a laxative in the pharmaceutical industry, fermentative production of organic acids	[19,47,53,57,58]

**Table 2:** Methane yield of some seaweed species. VS, volatile solids.

Seaweed Species	Methane Yield (mL·g^−1^ VS)	Reference
*Ascophyllum*	110	[98]
*Gracilaria*	280–400	[90,91]
*Laminaria* sp.	180–300	[53,92]
*Macrocystis pyrifera*	180–430	[85,88]
*Sargassum*	120–190	[99]
*Ulva lactuca*	200–480	[53,100]

**Table 3:** Pretreatment methods employed to improve the digestion of marine macroalgae and to increase the biochemical methane potential (BMP) to obtain a high yield of biogas.

Pretreatment Method	Technique	Description	Examples	Increase of BMP	Reference
Physical	Mechanical	Substrate fragmentation using manual knife mills, shredders or automatic hammer mills	*Laminaria digitata*, *Laminaria saccharina*, *Laminaria hyperborea*	+20%–50%	[105,109,113,114,115]
	Thermal	Heating at 125 °C–190 °C under pressure for up to an hour	*Saccharina latissima*	n.s.	[86,103,108]
Chemical	Alkaline	Alkali pretreatment, e.g., sodium hydroxide	*Ulva* spp.	+27%	[116]
	Acidic	Pretreatment with organic acids (citric acid, lactic acid, acetic acid, oxalic acid) or inorganic acids (e.g., hydrochloric acid, sulfuric acid)	*Laminaria digitata*, *Saccharina latissima*, *Fucus vesiculosus*	+4%	[102,117]
Biological	Microbial digestion	Aerobic microbial digestion (e.g., polysaccharide hydrolyzing bacteria, methanogenic archaea) or anaerobic digestion in one- or two-stage bioreactors	*Laminaria japonica*, *Laminaria hyperborean*, *Saccharina latissima*	n.s.	[63,77,118]
	Enzymatic digestion	Co-digestion with individual enzymes (e.g., pectinase, cellulase, hemicellulase, alginate lyase or protease) or with enzyme mixtures	*Furcellaria lumbricalis*, *Fucus vesiculosus*, *Palmaria palmate Laminaria digitata*, *Saccharina latissima*	+2%	[117,119]
Combined processes	Steam explosion	Thermal pretreatment at 160 °C–220 °C combined with a sudden drop in pressure.	*Saccharina latissima*	+20%	[103,110]
	Thermo-chemical	Thermal treatment (60 °C–220 °C) combined with the addition of different kinds of acidic or alkali reagents.	*Palmaria palmate*, *Fucus vesiculosus*	+10%–140%	[86,102,103]
	Biochemical	Both acidic (2.5% citric acid) and enzymatic (cellulase) pretreatments are applied to the substrate.	*Laminaria digitata*	+7%	[117]

**Table 4:** Worldwide production of major species of marine macroalgae in millions of tons. Source: Food and Agriculture Organization of the United Nations (2012) [44].

Continent	2008	2009	2010	2011
Africa	0.12	0.11	0.14	0.14
Americas	0.03	0.09	0.01	0.02
Asia	15.73	17.14	18.84	20.80
Europe	0.00	0.00	0.00	0.00
Oceania	0.00	0.01	0.01	0.01
Total	15.9	17.4	19.0	21.0
Year-on-year growth rate	5.9%	9.3%	9.5%	10.4%

**Table 5:** Selection of the type, quantity and origin of industrial and eutrophic macroalgal waste products available for anaerobic digestion.

Type of Waste	Source	Organism	Quantity	Composition	CH_4_ Potential	Reference
“Macroalgae meal”	Residue from agar-agar extraction	*Gelidium sesquipedale*	2000–2400 kg/day (dry powder)	High: carbon, nitrogen, hydrogen Low: ash	n.s.	[181]
“*Macrocystis pyrifera* residue”	Qingdao Mingye Seaweed Industrial Co. Ltd. (China)	*Macrocystis pyrifera*	n.s.	Moisture: 9.8% Ash: 59.2% VS: 18.3% Fixed carbon: 12.71% cellulose, hemicellulose, lignin	n.s.	[182]
*Gracilaria gracilis* residues	Residues from phycobiliprotein extraction	*Gracilaria gracilis*	n.s.	75% VS 21% ash 4% fixed carbon	n.s.	[183]
*Laminaria japonica* residues	Remains from industrial biomass processing	*Laminaria japonica*	50,000 t/year (wet mass); remains of 10%–30% biomass from downstream processing	50.9% VS 39.2% carbohydrate 11.4% protein 0.3% lipid 49.1% ash	172–214 mL·g^−1^·VS	[63]
Fermentation residue, saccharification residue	Residues from algal bioethanol production	*Gelidium Amani*	n.s.	Galactose: 52.4% Protein: 15.6% Cellulose: 14.9% Ash: 5.7% Others: 11.4%	239–283 mL·g^−1^·VS	[184]
Alginate extraction residues	Kelco/AIL factory at Barcaldine (Scotland)	*Ascophyllum* spp.**	n.s.	n.s.	198–237 mL·g^−1^·VS	[178]
Alginate extraction sludge	Protan A/S, Haugesund, Norway	*Laminaria hyperborea*, *Ascophyllum nodosum*	n.s.	78.8%–85.4% VS	70–280 mL·g^−1^·VS	[179]
Green tide 2008	Qingdao algae bloom (China)	*Enteromorpha prolifera*	150,000–1 million t (wet mass)	Moisture: 4.85% Ash: 17,63% VS: 70.29% Fixed carbon: 7.4%	n.s.	[154,165]
Macroalgae bloom	Venice lagoon (Italy), bloom	*Ulva rigida*, *Gracilaria confervoides*	40,000 t/year (wet mass)	25.4% total solids (TS) 32.0% VS	129–212 mL·g^−1^·VS	[76,96,185]
Green tide	Patagonia beaches (Chile), bloom	*Green seaweed*	8000 t/year (wet mass)	n.s.	n.s.	[185]
Green tide	Brittany beaches (France), bloom	*Ulva* sp.	100,000 t/year (wet mass)	n.s.	91–200 mL·g^−1^·VS	[73,100]
Golden tide	Gulf of Mexico, bloom	*Sargassum natans, Sargassum fluitans*	1 million/year	n.s.	n.s.	[154]
Beached macroalgae	Orbetello lagoon (Italy), bloom	*Gracilariopsis longissima*, *Chaetomorpha linum*	5000 t/year (wet mass)	30% TS 47% VS	380 mL·g^−1^·VS	[74]

**Table 6:** The selection of by- and co-products that can be extracted from seaweeds and their value [53].

Product	Content (% of Dry Weight)	Value (€/t Dry Weight)
Alginate	23	1265
Mannitol	12–21	645
Iodine	0.45	58.50
Potash	9.5	5.10
Phosphorous	0.3	2.70
